# Prenatal and Childhood Growth, and Hospitalization for Alcohol Use Disorders in Adulthood: The Helsinki Birth Cohort Study

**DOI:** 10.1371/journal.pone.0087404

**Published:** 2014-01-29

**Authors:** Jari Lahti, Marius Lahti, Anu-Katriina Pesonen, Kati Heinonen, Eero Kajantie, Tom Forsén, Kristian Wahlbeck, Clive Osmond, David J. P. Barker, Johan G. Eriksson, Katri Räikkönen

**Affiliations:** 1 Institute of Behavioural Sciences, University of Helsinki, Helsinki, Finland; 2 National Public Health Institute, Helsinki, Finland; 3 Medical Research Council Lifecourse Epidemiology Unit, University of Southampton, United Kingdom; 4 Department of General Practice and Primary Health Care, University of Helsinki; The National Institute for Health Innovation, New Zealand

## Abstract

**Background:**

Small birth size - an indicator of a sub-optimal prenatal environment - and variation in growth after birth have been associated with non-communicable diseases in later life. We tested whether birth size or growth in childhood associated with the risk of hospital admission for alcohol use disorders (AUDs) from early to late adulthood.

**Methods:**

The sample comprised 6544 men and 6050 women born between 1934 and 1944 in Helsinki, Finland. Data on anthropometric measures were extracted from medical records and diagnoses of AUD from the Finnish Hospital Discharge Register and Causes of Death Register covering a 40-year period from 1969 to 2008.

**Results:**

Altogether 171 women (2.8%) and 657 men (10.0%) were diagnosed at a hospital with AUD. After adjusting for major confounders, shorter length at birth, shorter height up to two years of age, and lower weight at two years associated with hospitalization for AUD in women. In men, slower growth in height, particularly from 2 to 7 years, and slower weight gain from 7 to 11 years as well as shorter height and lower weight at 7 and 11 years associated with a diagnosis of AUD in men.

**Conclusions:**

Pre- and postnatal growth associates with the risk for AUD later in life differently in women than in men: the fetal period and infancy seem to be the sensitive periods for women, whereas those for men the occur from toddlerhood onwards.

## Introduction

In Western European societies, the lifetime prevalence of alcohol dependence is around 5% to 8% with rates four to five times higher for men than for women [1], [2]. Alcohol use ranks among the top causes of disease burden [3] and its impact in the developed world is projected to increase [4]. Since alcohol use can be an important causal factor in the development of a multitude of medical conditions [5], identifying early risk factors for alcohol use disorders (AUD) could help in preventive action.

Within the developmental origins of health and disease framework (DOHaD), the associations between small birth size or slower growth in childhood and health outcomes in later life have been attributed to suboptimal environmental conditions during sensitive time periods that alter the structure and function of key organs such as the brain [6]. These environmental conditions include psychosocial stress and malnutrition, which are amenable to intervention [6], [7].

Various non-communicable health conditions, such as hypertension and several psychiatric conditions, show high comorbidity with AUDs [5], [8]–[10] and have been linked to small body size at birth [7], [11]–[16] as well as growth patterns in childhood [7], [12], [15], [17], [18]. However, whether body size at birth or later in life associates with the risk for AUDs remains uncertain. In addition, the few existing studies show conflicting results and focus on those who were born at the extreme low end of the birth weight distribution [19]–[23] or on those who were obese in childhood [24]. Birth cohort studies have suggested that those born at the extreme low end of the birth weight distribution are at around 40% higher risk for alcohol abuse or substance-related disorders [19], [20] while one study showed an inverse gradient across decreasing birth weight [25]. Studies focusing on those born prematurely and with very low birth weight (<1500 grams; VLBW) suggest that VLBW is associated with *less frequent* alcohol consumption in young adulthood [21]–[23]. To our knowledge, no studies have examined the associations between body size or growth in childhood and subsequent AUDs in adulthood, and few studies point to such associations with respect to other psychiatric morbidity [24], [26], [27].

Our study aimed to investigate whether length of gestation, body size at birth and physical growth measured serially from birth to 11 years of age associate with hospitalization for or death due to AUDs in the Helsinki Birth Cohort Study (HBCS) sample. In addition to testing for linear effects, we examined whether nonlinear associations existed between birth size and hospitalization for AUDs. We also tested whether associations between physical growth and AUDs stem from comorbid mental disorders.

## Materials and Methods

### Subjects

The original study cohort comprised 13345 subjects who were born in Helsinki, Finland at one of the two public maternity hospitals, who visited child welfare clinics, and who received a Finnish personal ID number. Of these participants, we excluded 191 individuals because they moved abroad or died before data collection for the Finnish Hospital Discharge Register (HDR) began in 1969, 226 who were missing data on AUDs, and 334 who were missing data on childhood socioeconomic position (SEP). The cohort available for the analyses comprised 12 594 subjects (6544 men and 6050 women together comprising 94.3% of the original cohort). The excluded participants had an earlier year of birth (p<0.02), were more often women (p<0.004) and were lighter (p = 0.03) and thinner at birth (p<0.001), but showed no differences from the included participants in childhood SEP, length of gestation, placental weight, or length and head circumference at birth (p-values >0.05). Furthermore, 847 participants were excluded from the analysis of neonatal characteristics due to missing data on the length of gestation. The Ethics Committee of the National Public Health Institute and the Ethics Committee of the Helsinki and Uusimaa Hospital District approved the HBCS. The study has was conducted in adherence to the Declaration of Helsinki.

### Measures of neonatal characteristics and physical growth

Data on year of birth, weight (g), length (cm), head circumference (cm), placental weight (g), and date of the mother's last menstrual period were collected from the birth records. Data on monthly changes in weight (kg) and height (cm) from birth to 2 years and annual changes from 2 to 11 years were estimated from child welfare clinic and school health records as described previously [28]. From these measures we calculated the ponderal index at birth (kg/m^3^) and body mass index (BMI: kg/m^2^).

Our primary indicator of SEP in childhood was occupational status of the father. These data were derived from school, child welfare clinic, and birth records (inferred from the last two, respectively, if data from school records were not available) and were classified as low SEP (manual workers: 62.3%), middle SEP (lower middle class: 22.6%), and high SEP (upper middle class: 15.2%). We used highest maternal SEP as secondary indicator of childhood socio-economic environment due to higher number of those belonging to unclassified category (e.g. housewives or students). Of the mothers, 50.9%, 34.2%, 3.8%, 11.1% belonged to low SEP, middle SEP, high SEP, and other/unclassified SEP, respectively [29].

### Definition of hospitalization for alcohol use disorder (AUD) and of other mental disorders

Diagnoses were extracted from the HDR, which contained data on all hospitalizations in psychiatric and general hospitals in Finland between 1969 and 2008. The HDR also includes personal and hospital ID numbers, dates of hospital admission and discharge, and primary as well as up to three subsidiary diagnoses at discharge. The HDR is a valid and reliable tool for research [30], and studies have shown that diagnoses based on the HDR are more reliable than those based on medical examination or interview- or questionnaire-based measures, especially when a combined best-estimate diagnosis served as the validation criteria [31]. Accuracy of the HDR data with respect to AUDs proved high [32], and hospitalization for AUDs have served as an outcome in several studies [33], [34]. The HDR has also shown high validity with regard to psychotic disorders in general [31] and schizophrenia in particular [35]. We also identified AUDs as causes of death from the National Causes of Death-Register (CDR), which contains records of primary and subsidiary causes of death from all deaths in Finland.

Diagnoses were entered into the HDR and CDR according to the International Classification of Diseases, Eighth Revision (ICD-8) until 1986, according to the ICD-9 using the Diagnostic and Statistical Manual of Mental Disorders, Revised Third Edition (DSM-III-R) criteria until 1995, and according to the ICD-10 since 1996. In the current study, the primary diagnoses of alcohol intoxication (ICD-9: 3050A and ICD-10: F10.0) as well as the primary and subsidiary diagnoses of harmful alcohol use, alcohol dependence, and psychotic disorder due to alcohol abuse (ICD-8/9: 291, 303 and ICD-10 F10.1-F10.9) from either register served to index the AUDs. In our sample, we identified 746 and 34 AUD cases based solely on the HDR and CDR, respectively, and identified 48 based on both registers (6.6% of the total sample).

We examined the different AUDs together as one broad diagnostic outcome rather than as separate entities (such as alcohol dependence and harmful use of alcohol) to make our definition of AUDs more comparable with the definition of AUDs in the DSM-5, that was introduced in 2013. However, in correspondence with ICD-10, our definition of AUDs includes also the primary and subsidiary diagnoses of alcohol psychoses, amnesic syndrome due to alcohol use, and other and unspecified mental and behavioural disorders due to alcohol use, and the primary diagnoses of alcohol intoxification.

The primary and up to three subsidiary diagnoses of mental disorders other than those due to alcohol use were drawn with the following codes: 295–302, 304–305, 306.4–306.5, 306.8, 306.98, and 307 from ICD-8; 292, 295–298, 300–302, 304, 3051–3059, 3071A, 3074, 3075A–3075B, 3078A, 3079X, 3090A, 3092C–3099X, and 312 from ICD-9; and F11–F69 from ICD-10 [36].

### Statistical analyses

First, we used logistic regression analyses to test whether neonatal characteristics and body size or growth from birth to age 11 were associated with hospitalization for AUDs in adulthood. We tested for nonlinear associations between birth size and hospitalization for AUDs using a model that included a squared term of the variable centered around the grand mean together with a non-squared linear variable. We focused on measurements conducted at birth, at 6 months of age, and at 1, 2, 7, and 11 years of age to represent growth in the prenatal period (at birth), in infancy (up to 2 years), and in childhood (up to 11 years). Physical size measurements served as continuous variables to test for linear and nonlinear associations. All the neonatal characteristics and the measures on attained size at different ages were converted to z-scores by sex. Postnatal growth variables were standardized residuals from linear regression models of weight, height, and BMI where body size at each point in time was regressed on corresponding measures at all earlier points in time, creating completely uncorrelated residuals reflecting growth conditional on history (i.e. conditional growth) [37]. In Model 1, we adjusted for year of birth and SEP in childhood in all analyses, and in analyses of birth size, we further adjusted for length of gestation. In Model 2, we further adjusted for maternal SEP. To test whether associations between physical growth and AUDs are attributable to comorbid mental disorders, in Model 3 we adjusted for other mental disorders in addition to those variables in Model 1. In addition, we reran all the Model 1 analyses in a sample comprising only those with any mental disorder (i.e. contrasting those with AUDs to those with other mental disorders).

Although we found no statistically significant interactions between body size or growth by sex (p-values for interactions >0.08), we conducted all the analyses separately for men and women, since previous studies have found major sex differences in the prevalence of AUDs [2] and in factors influencing alcohol use [38] as well as sex-specific associations between body size or growth and mental disorders [11], [24], [26], [27], [39]–[47].

## Results

Table 1 presents neonatal and adulthood characteristics according to sex. In men, low childhood SEP associated with 46.1% higher odds (95% Confidence Interval [CI] 11.4–86.6%, p = 0.002) for AUDs than for high childhood SEP. We found no associations between childhood SEP and AUDs in women (p>0.54).

**Table 1:** Characteristics of the sample.

Characteristic	Males		Females	
	N	Mean (SD)	N	Mean (SD)
At Birth:				
Weight (kg)	6544	3.5 (0.5)	6050	3.3 (0.5) #
Length (cm)	6490	50.6 (1.9)	5995	49.9 (1.8) #
Body Mass Index (kg/m^2^)	6484	13.5 (1.2)	5992	13.4 (1.2) #
Ponderal index (kg/m^3^)	6490	26.7 (2.2)	5995	26.8 (2.2)
Head circumference (cm)	6474	35.3 (1.5)	5988	34.7 (1.4) #
Gestational age (days)	6420	279.0 (14.3)	5935	279.7 (13.7)#
Placental weight (g)	6520	651.2 (119.9)	6040	639.2 (118.9)#
At age 6 months:				
Height (cm)	6534	67.8 (2.3)	6043	66.1 (2.3) #
Weight (kg)	6539	7.9 (0.9)	6049	7.4 (0.8) #
Body Mass Index (kg/m^2^)	6535	17.2 (1.4)	6045	16.8 (1.4) #
At age 1 year:				
Height (cm)	6534	76.5 (2.6)	6043	74.8 (2.6) #
Weight (kg)	6539	10.5 (1.1)	6049	9.8 (1.0) #
Body Mass Index (kg/m^2^)	6535	17.9 (1.4)	6045	17.5 (1.4) #
At age 2 years:				
Height (cm)	6536	86.6 (3.2)	6044	85.5 (3.2) #
Weight (kg)	6542	12.4 (1.2)	6049	11.9 (1.2) #
Body Mass Index (kg/m^2^)	6538	16.7 (1.2)	6046	16.4 (1.2) #
At age 7 years:				
Height (cm)	4909	120.7 (4.9)	4521	119.9 (4.8) #
Weight (kg)	4917	22.5 (2.7)	4527	22.2 (2.9) #
Body Mass Index (kg/m^2^)	4903	15.5 (1.1)	4512	15.5 (1.2)
At age 11 years:				
Height (cm)	4814	141.3 (6.0)	4409	141.4 (6.4)
Weight (kg)	4817	33.6 (4.6)	4407	34.3 (5.7) #
Body Mass Index (kg/m^2^)	4809	16.8 (1.5)	4403	17.1 (1.9) #
Father's SEP				*
Low	4022 (61.5%)		3819 (63.1%)	
Middle	1469 (22.4%)		1372 (22.7%)	
High	1053 (16.1%)		859 (14.2%)	
Mother's SEP				
Low	3290 (56.4%)		3120 (58.2%)	
Middle	2299 (39.4%)		2008 (37.4%)	
High	247 (4.2%)		234 (4.4%)	
Other (e.g.housewives)	708 (10.8%)		688 (11.4%)	
In adulthood				
Hospitalization for alcohol use disorders (yes)	657 (10.0%)		171 (2.8%) #	
Hospitalization for other mental disorders (yes)	565 (8.6%)		506 (8.4%)	

* : p<0.05; #: p<0.001.

### Neonatal characteristics and hospitalization for AUDs

**Figure 1:**
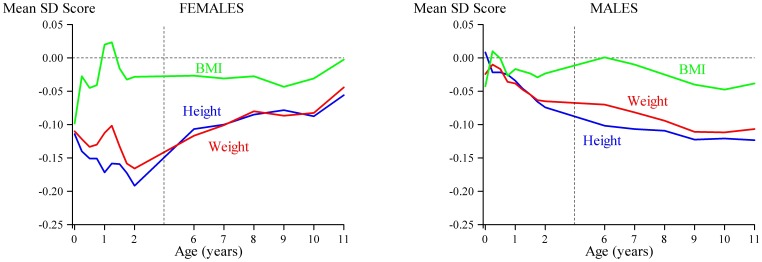
Trajectories of height (blue line), weight (red line), and BMI (green line) in those who were hospitalized for alcohol use disorder in adulthood. The mean values for height, weight and BMI of the monthly measures from birth to 2 years, and the yearly measures from 6 to 11 years, are set at zero with deviations from the mean expressed as standard deviations (*z* scores).

Table 2 shows that after adjusting for birth year, SEP in childhood and length of gestation, shorter length at birth increased the risk of hospitalization for AUDs linearly (p = 0.03) among women, and birth weight also shows a similar trend (p = 0.054). The ponderal index and head circumference at birth showed no association with the risk of hospitalization for AUDs in women (p-values>0.17; data not shown). Birth size showed nonlinear associations with the risk of hospitalization for AUDs in women (p-values >0.25). In men, we found neither linear (p-values >0.11) nor nonlinear (p-values >0.054) associations between body size at birth and the risk of hospitalization for AUDs. These associations are further illustrated in Figure 1. Moreover, we found no associations between gestational age and AUDs in men (linear: Odd's Ratio [OR]  = 1.04, 95% CI = 0.95 to 1.12, p = 0.39; non-linear: OR = 1.03, 95% CI = 0.98 to 1.09, p = 0.30) or in women (linear: OR = 1.03, 95% CI = 0.88 to 1.21, p = 0.70; non-linear: OR = 1.01, 95% CI = 0.91 to 1.13, p = 0.80). Further adjusting for maternal SEP (Model 2) or comorbid mental disorders (Model 3) changed none of the significance levels (Table 2), but restricting the sample to those with mental disorders rendered non-significant all associations between neonatal characteristics and AUDs in women (p-values >0.09) and in men (p-values >0.25).

**Table 2:** Odd's ratio (OR) of hospitalization for alcohol use disorders according to the decrease in standardized weight, length/height, and BMI from birth to 11 years of age.

Men								Women							
	Model 1					Model 2	Model 3		Model 1					Model 2	Model 3
	N	OR	95% CI		p	p	p	p	N	OR	95% CI		p	p	p
**Height/Length**															
Birth	6055	1.02	0.93	1.12	0.72	0.77	0.66	Birth	5609	1.22	1.02	1.46	0.03	0.03	0.05
6 months	6534	1.02	0.94	1.11	0.57	0.57	0.61	6 months	6043	1.18	1.02	1.38	0.03	0.03	0.05
1 year	6534	1.04	0.96	1.12	0.40	0.39	0.50	1 year	6043	1.20	1.03	1.40	0.02	0.02	0.02
2 years	6536	1.08	0.99	1.17	0.07	0.07	0.11	2 years	6044	1.22	1.05	1.42	0.01	0.01	0.01
7 years	4909	1.13	1.03	1.24	0.01	0.02	0.03	7 years	4521	1.12	0.95	1.33	0.18	0.21	0.30
11 years	4814	1.14	1.04	1.26	0.01	0.01	0.01	11 years	4409	1.07	0.90	1.27	0.44	0.49	0.64
**Weight**															
Birth	6094	1.06	0.97	1.17	0.21	0.25	0.18	Birth	5653	1.19	1.00	1.42	0.05	0.06	0.05
6 months	6539	1.03	0.95	1.11	0.55	0.55	0.88	6 months	6049	1.16	1.00	1.35	0.06	0.06	0.07
1 year	6539	1.04	0.96	1.13	0.32	0.33	0.44	1 year	6049	1.13	0.97	1.32	0.11	0.12	0.13
2 years	6542	1.07	0.99	1.16	0.10	0.11	0.14	2 years	6049	1.19	1.02	1.50	0.02	0.03	0.02
7 years	4917	1.10	1.00	1.20	0.05	0.07	0.09	7 years	4527	1.13	0.95	1.34	0.16	0.18	0.23
11 years	4817	1.13	1.03	1.24	0.01	0.01	0.03	11 years	4407	1.07	0.90	1.26	0.46	0.49	0.75
**BMI**															
Birth	6049	1.07	0.98	1.17	0.13	0.16	0.11	Birth	5606	1.12	0.95	1.33	0.17	0.18	0.15
6 months	6535	1.01	0.93	1.10	0.81	0.83	0.87	6 months	6045	1.05	0.90	1.23	0.51	0.51	0.50
1 year	6535	1.02	0.94	1.10	0.66	0.69	0.56	1 year	6045	0.99	0.85	1.15	0.87	0.86	0.85
2 years	6538	1.03	0.95	1.11	0.51	0.55	0.49	2 years	6046	1.04	0.89	1.21	0.65	0.66	0.55
7 years	4903	1.01	0.92	1.11	0.84	0.88	0.88	7 years	4512	1.05	0.88	1.24	0.61	0.61	0.56
11 years	4809	1.05	0.95	1.15	0.35	0.35	0.50	11 years	4403	1.02	0.86	1.21	0.79	0.79	0.97

CI: Confidence interval. All models are adjusted for year of birth and socioeconomic position in childhood, and models pertaining to body size at birth are also adjusted for gestational age (Model 1). In addition to those variables in Model 1, Model 2 and Model 3 are adjusted for maternal socioeconomic position or other mental disorders, respectively.

### Body size and conditional growth after birth and hospitalization for AUDs

Table 2 also shows that after adjusting for birth year and SEP in childhood, increased risk of hospitalization for AUDs was associated with shorter height from six months to two years in women (p-values<0.03). Moreover, in women, lower weight at two years increased the risk of hospitalization for AUDs (p = 0.02), and weight at six months showed a similar trend (p = 0.057). In men, shorter height and lower weight at 7 and 11 years associated with increased risk of hospitalization for AUDs (p-values<0.05; Figure 1). Further adjusting for maternal SEP (Model 2) or comorbid mental disorders (Model 3) did not change the significance levels (Table 2), except for one: in men, the association between lower weight at seven years and AUDs became non-significant in both models (p-values >0.07). Restricting the sample to those with mental disorders had no effect on the significance levels of the associations between body size after birth and AUDs in women (p-values<0.04), whereas in men, it rendered all associations non-significant (p-values >0.21).

We then tested whether growth conditional on history associated with hospitalization for AUDs. Among men we found that after adjusting for birth year and SEP in childhood, hospitalization for AUDs was associated with slower growth in height from two to seven years (OR = 1.10, 95% CI = 1.00 to 1.22, p = 0.048) and with slower weight gain from seven to 11 years (OR = 1.12, 95% CI = 1.01 to 1.23, p = 0.03), while weight gain from two to seven years showed a similar trend (OR = 1.10, 95% CI = 1.00 to 1.21, p = 0.06). We found no significant associations between growth conditional on history and AUDs in women. Further adjusting for maternal SEP (Model 2) or comorbid mental disorders (Model 3) yielded only one change in significance levels: in men, slower growth in height from two to seven years became non-significant (Model 2: OR = 1.1, 95% CI = 1.00 to 1.22, p = 0.06; and Model 3: OR = 1.09, 95% CI = 1.00 to 1.21, p = 0.09). Restricting the sample to those with mental disorders rendered all significant associations non-significant (p-values >0.19).

## Discussion

We explored whether length of gestation and body size or physical growth up to 11 years of age associated with hospitalization for AUDs in adulthood in 12594 women and men participating in the Helsinki Birth Cohort Study. Different growth periods were associated with increased risk for AUDs in both women and men. After adjusting for gestational age, birth year, and SEP in childhood, shorter length at birth associated linearly with increased risk of hospitalization for AUDs in women. This association was unattributable to maternal SEP or comorbid other mental disorders, although restricting the sample to those with any mental disorders rendered the association non-significant. AUDs showed no association with growth after birth, although women who were hospitalized for AUDs remained on average shorter up to two years of age, and weighed less at two years of age. These results were unattributable to maternal SEP or comorbid mental disorders. In men, birth characteristics showed no association with the risk for AUDs. However, men who were hospitalized because of AUDs had slower growth in height between two and seven years of age; they also gained less weight from seven to 11 years and, as a consequence, were shorter and weighed less at age 7 and 11 years. Similar findings related to weight and height are likely the result of slower growth in height, as there was no relationship with BMI in childhood. In men, maternal SEP or comorbid mental disorders had little effect on these associations, but restricting the sample to those with any mental disorders rendered the associations non-significant.

These results are in line with those of previous studies on population-based birth cohorts that have shown an inverse gradient between birth weight and substance-related disorders in young adulthood [25], and increased risk for AUDs [19] or substance-related disorders [20], [25]_ENREF_15 in those who were born with low birth weight [19] or were small for their gestational age [20], [25]. Our results add the previous literature by showing not only that the associations extend beyond birth weight, but also that slower intrauterine skeletal growth seemed to play a role in increasing the risk of hospitalization for AUDs in adulthood. Furthermore, the follow-up periods in previous research extend only into young adulthood, while our findings show that the effects persist across several decades into older adulthood. Our findings disagree with those found in young adults born prematurely: in previous studies, those born at VLBW reported less frequent heavy alcohol use or lower consumption of alcohol than did their peers born at term [21], [22]. However, these findings may be limited to those born the smallest and most immature, who were unlikely to survive when the subjects in the present cohort were born. As in this study, previous research has also shown that associations of birth size with mental health vary between men and women: some studies have reported stronger inverse associations between birth weight and emotional or attention problems in women than in men [40]–[43], while other studies show links between lower birth weight and depression in men, but not in women [44]–[47].

To the best of our knowledge, this study is the first to explore body size or physical growth in childhood with respect to AUDs in adulthood; consequently, the current findings cannot be directly compared to the previous ones. Cross-sectional studies on associations between body size and psychiatric morbidity show conflicting results, with some linking obesity with psychiatric problems [26], [48]–[51], and others showing no association [52] or an inverse association between BMI and mental disorders [53], [54]. Longitudinal studies suggest sex-specificity in associations between childhood body size or physical growth and psychiatric morbidity in later life [24], [26], [27]. In girls, but not in boys, higher BMI and gain in BMI in childhood linked with anxiety disorders and depression in adulthood [26]. Moreover, chronic obesity in childhood increased the risk for depression in adolescence only in boys, but for oppositional disorder in both sexes [24]. Another study also found no association between childhood overweight and subsequent depression [52]. Furthermore, still other studies have shown that these associations may be not only sex-specific, but also specific to a particular growth period. Physical growth in infancy and in childhood show different patterns with respect to risk of hospitalization for personality disorders in adulthood in both boys and girls [27]. Thus, some of the inconsistencies in the findings may stem from a lack of physical growth data on specific age periods.

Our finding that restricting the sample to those with mental disorders attenuated many of the associations is in line with earlier findings that smaller birth size associates with a wide range of mental disorders [25] and may therefore be considered a marker more for overall vulnerability than for a specific disorder. However, our findings that in women shorter height in early childhood and lower weight at two years associated with increased risk of hospitalization for AUDs after adjusting for other mental disorders and after restricting the sample to those with any mental disorder, suggest that these associations may be more specific and confined to AUDs.

Our results cannot determine the biological and psychosocial mechanisms that underlie the associations between smaller body size and the risk for AUDs. The DOHaD framework postulates that these conditions may relate to environmental psychosocial stress and/or malnutrition that alter the structure and function of the brain [6], [7]. Indeed, in animal studies, prenatal stress [55], [56] and the prenatal administration of synthetic glucocorticoids [57], probably mediated via hypothalamic pituitary adrenocortical (HPA) axis activity, have associated with a variety of alterations in hormonal axes, in monoamine neurotransmission, and in the morphology of the offspring [56]. In addition, human studies have linked several indicators of extreme small birth size with structural or functional alterations in the brain. Greater birth weight within the normal range was recently linked with generalized increase in brain volume, which in the cortical sheet, seems to be driven by increased surface area [58] as well as with increase in the volume of several striatal areas [59]. Similar results were found in monozygotic twins, suggesting that environmental factors underlie these associations [58], and in singletons [59]. Intrauterine growth retardation has been linked with metabolic disturbances in brain serotonin synthesis [60], low birth weight with increased lateral ventricular volume [61], and preterm birth with a smaller hippocampus [62], [63]. Moreover, a recent meta-analysis showed children who were very premature and/or of very low birth weight to have significantly smaller total brain volume than term-born comparison group, less white and grey matter volume, and smaller cerebellum, hippocampus, and corpus callosum [64]. Roza et al. [65] showed that reduced fetal growth in head circumference and biparietal diameter predicted lower ventricular volume in infancy. Interestingly, previous studies have implicated serotonin genes in alcohol dependence [66] and have linked alcohol use with alterations in HPA axis activity [67] and hippocampal volume [68] in adolescents.

Our findings suggest that sensitive periods with respect to the risk for AUDs may differ for men and women. Exposures *in utero* and in infancy may have greater effects for women, whereas for men, the sensitive periods occur later, from toddlerhood onwards. Consistent with these results, exposure to prenatal stress associated with larger alterations in monoamine neurotransmission [69] and HPA axis function [70], and increased anxiety-like behaviors [71] in female animals, whereas males exhibited more learning deficits [71].

The strengths of our study are its longitudinal study design, its relatively large sample of participants derived from the general population, and its use of comprehensive register data. Moreover, we measured growth at several points in time, which permitted us to track critical periods of growth with respect to subsequent AUDs. Our study also has its limitations. First, we have no data on parental mental disorders or risk behaviors such as alcohol use or smoking. Since smoking or alcohol use during pregnancy may increase the risk for small birth size [72] as well as the risk for psychiatric disorders in the offspring [73], [74], the associations observed may have resulted from prenatal exposures. Moreover, the lack of data on parental alcohol use after birth for our sample precluded us from investigating and accounting for the role of social learning in the development of AUDs. Second, our data on diagnoses were based on hospital records and not everyone with AUD requires hospitalization. Moreover, the HDR data do not cover the years between 1934 and 1969. Consequently, participants with AUDs may have had severe disorders, so our results may not be generalized to less severe disorders. However, the proportion of participants in our sample diagnosed with AUDs (6.6%) is only slightly lower than the lifetime prevalence of 7.9% reported recently in a population-based sample of Finns over 30 years of age [1]. Third, although our participants correspond to 94.3% of the original cohort, one limitation of the external validity of our findings is that they do not include those who died or emigrated before assignment of personal ID numbers and those who did not attend free-of-charge, voluntary child welfare clinics. Moreover, loss to follow-up is inevitable. Compared with the original cohort, participation was unrelated to childhood SEP, length of gestation, placental weight, or length and head circumference at birth, but the participants of this study were born later, were more often men, and tended to be heavier at birth. These differences could, however, introduce bias only if the associations between growth and AUDs in people included in the sample differed from such associations in those who were not. Furthermore, one should be cautious about generalizing our findings to people living in other types of societies or to those born in later decades.

In summary, the associations between anthropometric traits, indicators of the prenatal and childhood nutritional and psychosocial environment, and AUDs showed differences between women and men: shorter length/height at birth and in infancy in girls and in childhood in boys, associated with increased risk for AUDs in later life.
